# Characteristics of small breast and/or ovarian cancer families with germline mutations in *BRCA1* and *BRCA2*

**DOI:** 10.1038/sj.bjc.6690235

**Published:** 1999-03

**Authors:** M J L Ligtenberg, F B L Hogervorst, H W Willems, P J W Arts, G Brink, S Hageman, E A J Bosgoed, E Van der Looij, M A Rookus, P Devilee, E M A W Vos, G Wigbout, P M Struycken, F H Menko, E J Th Rutgers, E H Hoefsloot, E C M Mariman, H G Brunner, L J Van't Veer

**Affiliations:** Department of Human Genetics, University Hospital Nijmegen, PO Box 9101, Nijmegen, 6500 HB Nijmegen, The Netherlands; Department of Pathology, University Hospital Nijmegen, PO Box 9101, Nijmegen, 6500 HB Nijmegen, The Netherlands; Department of Pathology and The Family Cancer Clinic, The Netherlands Cancer Institute, Plesmanlaan 121, Amsterdam, 1066 CX Amsterdam, The Netherlands; Department of Epidemiology, The Netherlands Cancer Institute, Plesmanlaan 121, Amsterdam, 1066 CX Amsterdam, The Netherlands; Department of Human Genetics and Department of Pathology, University of Leiden, PO Box 9503, 2300 RA Leiden, The Netherlands; Family Cancer Clinic, Department of Clinical Genetics, PO Box 7057, Amsterdam, 1007 MB Amsterdam, The Netherlands

**Keywords:** breast cancer, ovarian cancer, bilateral breast cancer, *BRCA1*, *BRCA2*

## Abstract

For families with a small number of cases of breast and/or ovarian cancer, limited data are available to predict the likelihood of genetic predisposition due to mutations in *BRCA1* or *BRCA2*. In 104 families with three or more affected individuals (average 3.8) seeking counselling at family cancer clinics, mutation analysis was performed in the open reading frame of *BRCA1* and *BRCA2* by the protein truncation test and mutation-specific assays. In 31 of the 104 families tested, mutations were detected (30%). The majority of these mutations (25) occurred in *BRCA1*. Mutations were detected in 15 out of 25 families (60%) with both breast and ovarian cancer and in 16 out of 79 families (20%) with exclusively cases of breast cancer. Thus, an ovarian cancer case strongly predicted finding a mutation (*P* < 0.001). Within the group of small breast-cancer-only families, a bilateral breast cancer case or a unilateral breast cancer case diagnosed before age 40 independently predicted finding a *BRCA1* or *BRCA2* mutation (*P* = 0.005 and *P* = 0.02, respectively). Therefore, even small breast/ovarian cancer families with at least one case of ovarian cancer, bilateral breast cancer, or a case of breast cancer diagnosed before age 40, should be referred for mutation screening. © 1999 Cancer Research Campaign


*British Journal of Cancer* (1999) **79**(9/10), 1475–1478
© 1999 Cancer Research Campaign
Article no. bjoc.1998.0235

### Characteristics of small breast and/or ovarian cancer families with germline mutations in *BRCA1* and *BRCA2*

**MJL Ligtenberg[1,2], FBL Hogervorst[3], HW Willems[2], PJW Arts[1], G Brink[3], S Hageman[3], EAJ Bosgoed[1], E Van der Looij[1], MA Rookus[4], P Devilee[5], EMAW Vos[3], G Wigbout[3], PM Struycken[3], FH Menko[3,6], EJTh Rutgers[3], EH Hoefsloot[1], ECM Mariman[1], HG Brunner[1] and LJ Van't Veer[3]**

Departments of [1]Human Genetics and [2]Pathology, University Hospital Nijmegen, PO Box 9101, 6500 HB Nijmegen, The Netherlands; [3]Department of Pathology and The Family Cancer Clinic, and [4]Department of Epidemiology, The Netherlands Cancer Institute, Plesmanlaan 121, 1066 CX Amsterdam, The Netherlands; [5]Department of Human Genetics and Department of Pathology, University of Leiden, PO Box 9503, 2300 RA Leiden, The Netherlands; [6]Family Cancer Clinic, Department of Clinical Genetics, University Hospital Vrije Universiteit, PO Box 7057, 1007 MB Amsterdam, The Netherlands

**Summary** For families with a small number of cases of breast and/or ovarian cancer, limited data are available to predict the likelihood of genetic predisposition due to mutations in *BRCA1* or *BRCA2*. In 104 families with three or more affected individuals (average 3.8) seeking counselling at family cancer clinics, mutation analysis was performed in the open reading frame of *BRCA1* and *BRCA2* by the protein truncation test and mutation-specific assays. In 31 of the 104 families tested, mutations were detected (30%). The majority of these mutations (25) occurred in *BRCA1*. Mutations were detected in 15 out of 25 families (60%) with both breast and ovarian cancer and in 16 out of 79 families (20%) with exclusively cases of breast cancer. Thus, an ovarian cancer case strongly predicted finding a mutation ($P < 0.001$). Within the group of small breast-cancer-only families, a bilateral breast cancer case or a unilateral breast cancer case diagnosed before age 40 independently predicted finding a *BRCA1* or *BRCA2* mutation ($P = 0.005$ and $P = 0.02$, respectively). Therefore, even small breast/ovarian cancer families with at least one case of ovarian cancer, bilateral breast cancer, or a case of breast cancer diagnosed before age 40, should be referred for mutation screening.

**Keywords:** breast cancer; ovarian cancer; bilateral breast cancer; *BRCA1*; *BRCA2*

Breast cancer is the most common malignancy in women. A family history of breast cancer has long been recognized as one of the strongest risk factors for the disease and it is estimated that 5–10% of all breast cancer cases can be attributed to inherited autosomal dominant susceptibility genes. Two genes involved in hereditary breast and/or ovarian cancer syndromes, *BRCA1* and *BRCA2*, were cloned (Miki et al, 1994; Wooster et al, 1995; Tavtigian et al, 1996). Initially, reports on extended high-risk families suggested that germline mutations in *BRCA1* would account for about 45% of the hereditary site-specific breast cancer families, and for more than 80% of the hereditary breast and ovarian cancer families (Easton et al, 1993). Similar studies predicted the involvement of *BRCA2* in the majority of non-*BRCA1*-linked breast cancer families (Wooster et al, 1994). Recent studies, however, show that germline mutations in *BRCA1* and *BRCA2* are only associated with approximately half of all hereditary breast and/or ovarian cancer, and that this proportion varies widely among populations (for review see Szabo and King, 1997).

Most of our current knowledge on genetic predisposition by *BRCA1* or *BRCA2* is based on families with multiple cases of breast and/or ovarian cancer. Few data are available on less extended families, i.e. with only three to five individuals with breast and/or ovarian cancer, although they form the majority of families seeking genetic advice in the family cancer clinics.

In this study we determined the frequency of both *BRCA1* and *BRCA2* germline mutations in relatively small families whose cancer risk was evaluated at our family cancer clinics. Striking differences in the prevalence of *BRCA1* or *BRCA2* mutations were found in families with only breast cancer when families were classified according to the presence or absence of bilateral breast cancer and the age of diagnosis of the youngest patient.

*Received 19 May 1998*
*Revised 7 August 1998*
*Accepted 20 August 1998*

*Correspondence to:* MJL Ligtenberg, Department of Human Genetics, University Hospital Nijmegen, PO Box 9101, 6500 HB Nijmegen, The Netherlands

### MATERIALS AND METHODS

### Study population

Self-referred or physician-referred breast and/or ovarian cancer-prone families visiting the family cancer clinics at The University Hospital Nijmegen and The Netherlands Cancer Institute were the subjects of this study. For the categorization of the families, first-, second- and third-degree relatives of the person tested were taken into consideration. The number of affected individuals varied between three and nine (mean 4.3; median 4) in families with at least one case of ovarian cancer and between three and five (mean of 3.6; median 3) in families without ovarian cancer. If possible, the patients with the highest prior probability of carrying a mutation in one of the susceptibility genes were examined. In 93 families one or more patients with a history of breast and/or ovarian cancer were investigated. In 11 families only unaffected first-degree relatives could be sampled for various reasons. On average, 1.4 individuals were tested in each family. For most patients clinical archives and pathological records were retrieved and re-evaluated. All analyses were performed after pretest counselling and after informed consent had been obtained.

### Mutation detection in *BRCA1* and *BRCA2*

Both labs performed the complete mutation screening for the families referred to their clinics. Isolation of DNA and RNA from EDTA blood and generation of polymerase chain reaction (PCR) and nested reverse transcription (RT)-PCR products were performed using standard procedures. Primers, containing a T7 promoter, a eukaryotic translation initiation sequence and gene-specific sequences, were used to generate PCR products suitable for analysis by the protein truncation test (PTT). PCR products were in vitro transcribed and translated in the TNT/T7 coupled reticulolysate system (Promega), essentially as described by the manufacturer. $^{35}$S-methionine (NKI/AvL lab) or biotinylated tRNA-Lysine (tRNA-scend, Promega) (Nijmegen lab) were used to label de novo synthesized proteins.

For PTT analysis of *BRCA1*, five or six overlapping fragments covering the entire open reading frame were amplified essentially as described before (Hogervorst et al, 1995). The 5′ and 3′ coding exons (exons 2, 3, 5, 6, 23 and 24) as well as exon 20 were screened by the Nijmegen laboratory for the presence of frame-shift mutations in multiplex PCRs using fluorescently labelled primers annealing in the surrounding introns. The Netherlands Cancer Institute laboratory screened for the 185delAG recurrent mutation in exon 2 by single base sequencing. The frequently occurring deletions of exon 13 and 22 were determined by PCR analysis of genomic DNA essentially as described (Petrij-Bosch et al, 1997).

For the *BRCA2* mutation analysis by PTT, nine overlapping fragments encompassing the entire open reading frame were designed. Four primer sets were used to amplify exon 11 from genomic DNA (fragments C, D, E and F). The remaining fragments (two fragments (A and B) upstream and including the 5′ region of exon 11, three fragments (G, H, K) downstream and including the 3′ region of exon 11) were amplified from cDNA by nested PCR. PTT analysis of fragment H (encompassing exons 15–23) failed for most patients. In about 10% of individuals the interpretation of the PTT of fragment K (exons 19–27) was complicated due to the insertion of an alternatively spliced exon (exon 20b), which generates a frame-shift and a premature stop (data not shown). As a control for the PTT on RT-PCR products, an additional primer set was designed to analyse exon 10 from genomic DNA by PTT (all primer sequences are available upon request).

**Table 1** *BRCA1* and *BRCA2* mutations

| Mutation | Change | No. of families |
|---|---|---|
| *BRCA1* | | |
| 185delAG | stop 39 | 1 |
| 1438delT | stop 440 | 1 |
| 2312del5 | stop 736 | 6 |
| 2804delAA | stop 901 | 5 |
| 3109insAA | stop 999 | 1 |
| 3604delA | stop 1209 | 2 |
| 3867G->T | E1250X | 2 |
| IVS21-36 del510 (del exon 22) | stop 1803 | 8 |
| *BRCA2* | | |
| 690delAA | stop 157 | 1 |
| 5823delAT | stop 1874 | 1 |
| 6174delT | stop 2003 | 1 |
| 6503delTT | stop 2099 | 2 |

### Direct sequence analysis

Direct sequence analysis was performed using the dye primer or the *Taq* DyeDeoxy terminator cycle sequencing kit (Applied Biosystems and Perkin Elmer) according to the manufacturer. The sequence reactions were run and analysed using an automatic sequencer (ABI 373A).

**Table 2** Predictive factors of finding a *BRCA1* or *BRCA2* mutation in small families with breast and/or ovarian cancer

| Type of family | No. of families | No. of *BRCA1* mutations | No. of *BRCA2* mutations | Total no. of mutations | Test of the factor predicting mutation status[a] (*P*-value) | |
|---|---|---|---|---|---|---|
| | | | | | Univariate model | Multivariate model |
| Total group | 104 | 26 | 5 | 31 (30%) | | |
| Only breast cancer | 79 | 11 | 5 | 16 (20%) | | |
| Breast and ovarian cancer | 25 | 15 | 0 | 15 (60%) | < 0.001 | < 0.001 |
| Breast and ovarian cancer[b] | | | | | | |
| ≥ 2 breast with 1 ovarian | 14 | 8 | 0 | 8 (57%) | | |
| ≥ 1 breast with 2 ovarian | 11 | 7 | 0 | 7 (64%) | = 0.806 | |
| Only breast cancer[c] | | | | | | |
| No bilateral | 43 | 2 | 1 | 3 (7%) | | |
| ≥ 1 bilateral | 36 | 9 | 4 | 13 (36%) | = 0.004 | = 0.005 |
| No patient < 40 years | 33 | 0 | 2 | 2 (6%) | | |
| ≥ 1 patient < 40 years | 46 | 11 | 3 | 14 (30%) | = 0.016 | = 0.020 |
| < 3 patients < 50 years | 45 | 2 | 4 | 6 (13%) | | |
| ≥ 3 patients < 50 years | 34 | 9 | 1 | 10 (29%) | = 0.085 | = 0.863 |

[a]*P*-value of the β-coefficient of the factor in a logistic model with mutation status as the dependent variable: the multivariate models include (1) the presence of ovarian cancer, ≥ 1 bilateral cancer and ≥ 1 breast or ovarian cancer below age 40, for the total group; and (2) the three factors listed for the group with only breast cancer. [b]At least 3 cases/family, mean 4.3 cases/family, median 4 cases. [c]A total of 3–5 cases/family, mean 3.6 cases/family, median 3 cases.

*© Cancer Research Campaign 1999*

**Table 3** Characteristics of families with three to five cases of breast cancer without ovarian cancer

| Type of breast-cancer-only family | No. of families | No. of *BRCA1* mutations | No. of *BRCA2* mutations | Total no. of mutations |
|---|---|---|---|---|
| No bilateral breast cancer | | | | |
| No patient < 40 years | 20 | 0 | 0 | 0 (0%) |
| ≥ 1 patient < 40 years | 23 | 2 | 1 | 3 (14%) |
| At least one bilateral breast cancer | | | | |
| No patient < 40 years | 13 | 0 | 2 | 2 (14%) |
| ≥ 1 patient < 40 years | 23 | 9 | 2 | 11 (48%) |

### Statistical analysis

In order to predict the finding of a *BRCA1* or *BRCA2* mutation, logistic models were fitted with mutation status as the dependent variable and the various family cancer histories as potential predicting factors. In multivariate models we investigated mutual dependency of the predicting factors. The reported *P*-values for the tests of significance of the regression coefficients are two-sided.

### RESULTS

### Mutation analysis of *BRCA1* and *BRCA2*

In individuals from 104 families with at least three members affected with breast and/or ovarian cancer, the open reading frames of *BRCA1* and *BRCA2* were screened for mutations. In 26 families a mutation in *BRCA1* and in five families a mutation in *BRCA2* was detected. All mutations are listed in Table 1.

### Classification of families and variables predicting their mutation frequencies

To analyse in which kinds of families the mutations were found, all families were first categorized according to the presence or absence of one or more cases of ovarian cancer (Table 2). In 15 out of 25 families (60%) with at least one case of ovarian cancer, a mutation was detected. In 20% (16/79) of the families with three to five cases of breast cancer without ovarian cancer a mutation was found. For the total group the presence of ovarian cancer strongly predicted mutation status (univariate and multivariate $P < 0.001$; Table 2). Among families with ovarian cancer the number of ovarian cancer cases (one or more) was not predictive, but the subgroups were rather small ($P = 0.806$; Table 2). The proportion of mutation-positive families was similar for families with three affected cases (14/49, 29%) as compared to families with more cases (17/55, 31%; $P = 0.795$; data not shown).

The families without a case of ovarian cancer, the so-called 'breast-cancer-only' families, were classified according to the presence of bilateral breast cancer, the presence of at least one case diagnosed before age 40, or the number of patients diagnosed before age 50. Table 2 shows that the probability of finding a mutation was markedly higher in the families with at least one case of bilateral breast cancer (36%) than in families with only unilateral breast cancer (7%) ($P = 0.004$). Also, the presence of at least one breast cancer case before age 40 strongly predicted finding a *BRCA1* or *BRCA2* mutation within these breast-cancer-only families. A mutation was found in 30% of the families with such a case and in only 6% of the remaining families ($P = 0.016$). The number of cases diagnosed before age 50 was less predictive for mutation status ($P = 0.085$; Table 2).

The presence of a bilateral case, and the presence of a case younger than 40, proved to be independent predicting factors in the multivariate model ($P = 0.005$ and $P = 0.020$, respectively; Table 2), which means that within families with a bilateral breast cancer case, the probability of finding a mutation was even higher if at least one case was diagnosed before age 40. Table 3 shows that a mutation was found in 11 out of 23 such families (48%). In contrast, in all 20 families with exclusively unilateral breast cancer diagnosed after age 40, no mutation was found.

### DISCUSSION

Since the cloning of *BRCA1* and *BRCA2* it has become possible to offer women DNA tests for the two most common breast cancer susceptibility genes without depending on linkage analyses. The present study provides insight into the frequency of *BRCA1* and *BRCA2* mutations in relatively small cancer-prone families. We observed that most mutation-positive families share specific characteristics.

More than 90% of the mutations in *BRCA1* and *BRCA2* reported to the Breast Cancer Information Core electronic database (internet address: http://www.nhgri.nih.gov/Intramural_research/Lab_transfer/Bic/) should be detectable by the techniques used here. Thirty-one families (30%) were shown to have a germline mutation, either in *BRCA1* or *BRCA2*. All eight different *BRCA1* mutations have been found more than once, either in our study group or in other Dutch families (Hogervorst et al, 1995; Peelen et al, 1997; Petrij-Bosch et al, 1997), supporting the observation of strong founder effects in the Dutch population.

In 60% of families with at least one case of ovarian cancer, a mutation was found, showing that in small families the presence of ovarian cancer in combination with a family cancer history is a good indication for genetic predisposition. Our study confirms that the presence of a *BRCA1* mutation confers a high risk of developing ovarian cancer, since this cancer was present in 58% of the families with a *BRCA1* mutation.

In families without ovarian cancer, we found that the presence of at least one breast cancer case diagnosed before age 40 and the presence of one bilateral breast cancer case, are strong independent predicting factors for the presence of a mutation in *BRCA1* or *BRCA2* (Tables 2 and 3). A study on the frequency of *BRCA1* mutations only in a group of 798 persons at elevated risk of hereditary breast/ovarian cancer showed that the probability of having a *BRCA1* mutation is increased when the index patient has bilateral breast cancer in comparison to an index patient with unilateral

© Cancer Research Campaign 1999

breast cancer (Shattuck-Eidens et al, 1997). The predictive value of bilateral breast cancer was not noted in another set of small families (Couch et al, 1997). However, this discrepancy may be due to their inclusion of families with ovarian cancer in all analyses.

More than twice as many *BRCA1* as *BRCA2* mutations were found in families with exclusively breast cancer. However, in families with an age at diagnosis of the youngest patient of over 40 years, only *BRCA2* mutations were detected. These data support the theory that *BRCA2* mutations are an infrequent cause of early-onset breast cancer (Krainer et al, 1997), but that they are still of importance in familial late-onset breast cancer.

In the families in which no mutation was found, either a mutation in *BRCA1* and *BRCA2* that is not detectable by the techniques used (e.g. large genomic deletions, promoter or missense mutations) might still be present, a genetic susceptibility caused by mutations in another thus far unidentified gene might be present, or the clustering of breast and/or ovarian cancer might have occurred by chance.

The results of the screening for *BRCA1* and *BRCA2* mutations in 104 families indicate that there is a considerable risk of genetic predisposition in those small families with at least one case of ovarian cancer, bilateral breast cancer, or a case of breast cancer diagnosed before age 40. Therefore, even small breast cancer families with these characteristics should be referred for *BRCA1* and *BRCA2* mutation testing.

### ACKNOWLEDGEMENTS

The authors thank Drs CA van Asperen, I Kluyt, CM Aalfs, J van de Ende, C van Ravenswaaij, L Beex and the staff at the Familial Cancer Clinic Nijmegen for their cooperation in this study, M Siers for technical assistance, Dr A Geurts van Kessel for continuous support, and The Dutch Cancer Society and the Nijbakker–Morra Foundation for financial support.

### REFERENCES

Couch FJ, DeShano ML, Blackwood A, Calzone K, Stopfer J, Campeau L, Ganguly A, Rebbeck T and Weber BL (1997) *BRCA1* mutations in women attending clinics that evaluate the risk of breast cancer. *N Engl J Med* **336**: 1409–1415

Easton DF, Bishop DT, Ford D and Crockford GP (1993) Genetic linkage analysis in familial breast and ovarian cancer: results from 214 families. The Breast Cancer Linkage Consortium. *Am J Hum Genet* **52**: 678–701

Hogervorst FBL, Cornelis RS, Bout M, van Vliet M, Oosterwijk JC, Olmer R, Bakker B, Klijn JGM, Vasen HFA, Meijers-Heijboer H, Menko FH, Cornelisse CJ, den Dunnen JT, Devilee P and van Ommen GJB (1995) Rapid detection of *BRCA1* mutations by the protein truncation test. *Nat Genet* **10**: 208–212

Krainer M, Silva-Arrieta S, FitzGerald MG, Shimada A, Ishioka C, Kanamaru R, MacDonald DJ, Unsal H, Finkelstein DM, Bowcock A, Isselbacher KJ and Haber DA (1997) Differential contributions of *BRCA1* and *BRCA2* to early-onset breast cancer. *N Engl J Med* **336**: 1416–1421

Miki Y, Swensen J, Shattuck-Eidens D, Futreal PA, Harshman K, Tavtigian S, Liu Q, Cochran Ch, Bennett LM, Ding W, Bell R, Rosenthal J, Hussey Ch, Tran Th, McClure M, Frye Ch, Hattier T, Phelps R, Haugen-Strano A, Katcher H, Yakumo K, Gholami Z, Shaffer D, Stone S, Bayer S, Wray Ch, Bogden R, Dayannath P, Ward J, Tonin P, Narod S, Bristow PK, Norris FH, Helvering L, Morrison P, Rosteck P, Lai M, Barrett JC, Lewis C, Neuhausen S, Cannon-Albright L, Goldgar D, Wiseman R, Kamb A and Skolnick MH (1994) A strong candidate for the breast and ovarian cancer susceptibility gene *BRCA1*. *Science* **266**: 66–71

Peelen T, van Vliet M, Petrij-Bosch A, Mieremet R, Szabo C, van den Ouweland AWM, Hogervorst F, Brohet R, Ligtenberg MJL, Teugels E, van der Luijt R, van der Hout AH, Gille JJP, Pals G, Jedema I, Olmer R, van Leeuwen I, Newman B, Plandsoen M, van der Est M, Brink G, Hageman S, Arts PJW, Bakker MM, Willems HW, van der Looij E, Neyns B, Bonduelle M, Jansen R, Oosterwijk JC, Sijmons R, Smeets HJM, van Asperen CJ, Meijers-Heijboer H, Klijn JGM, de Greve J, King MC, Menko FH, Brunner HG, Halley D, van Ommen GJB, Vasen HFA, Cornelisse CJ, van't Veer LJ, de Knijff P, Bakker E and Devilee P (1997) A high proportion of novel mutations in *BRCA1* with strong founder effects among Dutch and Belgian hereditary breast and ovarian cancer families. *Am J Hum Genet* **60**: 1041–1049

Petrij-Bosch A, Peelen T, van Vliet M, van Eijk R, Olmer R, Drüsedau M, Hogervorst FBL, Hageman S, Arts PJW, Ligtenberg MJL, Meijers-Heijboer H, Klijn JGM, Vasen HFA, Cornelisse CJ, van't Veer LJ, Bakker E, van Ommen GJB and Devilee P (1997) *BRCA1* genomic deletions are major founder mutations in Dutch breast cancer patients. *Nat Genet* **17**: 341–345

Shattuck-Eidens D, Oliphant A, McClure M, McBride C, Gupte J, Rubano T, Pruss D, Tavtigian SV, Teng DHF, Adey N, Staebell M, Gumpper K, Lundstrom R, Hulick M, Kelly M, Holmen J, Lingenfelter B, Manley S, Fujimura F, Luce M, Ward B, Cannon-Albrigt L, Steele L, Offit K, Gilewski T, Norton L, Brown K, Schulz Ch, Hampel H, Schluger A, Giulotto E, Zoli W, Ravaioli A, Nevanlinna H, Pyrhonen S, Rowley P, Loader S, Osborne MP, Daly M, Tepler I, Weinstein PL, Scalia JL, Michaelson R, Scott RJ, Radice P, Pierotti MA, Garber JE, Isaacs C, Peshkin B, Lippman ME, Dosik MH, Caligo MA, Greenstein RM, Pilarski R, Weber B, Burgemeister R, Frank TS, Skolnick MH and Thomas A (1997) *BRCA1* sequence analysis in women at high risk for susceptibility mutations. Risk factor analysis and implications for genetic testing. *JAMA* **278**: 1242–1250

Szabo CI and King MC (1997) Population genetics of *BRCA1* and *BRCA2*. *Am J Hum Genet* **60**: 1013–1020

Tavtigian SV, Simard J, Rommens J, Couch F, Shattuck-Eidens D, Neuhausen S, Merajver S, Thorlacius S, Offit K, Stoppa-Lyonnet D, Belanger C, Bell R, Berry S, Bogden R, Chen Q, Davis T, Dumont M, Frye C, Hattier T, Jammulapati S, Janecki T, Jiang P, Kehrer R, Leblanc JF, Mitchell JT, McArthur-Morrison J, Nguyen K, Peng Y, Samson C, Schroeder M, Snyder SC, Steele L, Stringfellow M, Stroup C, Swedlund B, Swensen J, Teng D, Thomas A, Tran T, Tran T, Tranchant M, Weaver-Feldhaus J, Wong AKC, Shizuya H, Eyfjord JE, Cannon-Albright L, Labrie F, Skolnick MH, Weber B, Kamb A and Goldgar Dt (1996) The complete *BRCA2* gene and mutations in chromosome 13q-linked kindreds. *Nat Genet* **12**: 333–337

Wooster R, Neuhausen SL, Mangion J, Quirk Y, Ford D, Collins N, Nguyen K, Seal S, Tran T, Avrill D, Fields P, Marshall G, Narod S, Lenoir GM, Lynch H, Feunteun J, Devilee P, Cornlisse CJ, Menko FH, Daly PA, Ormiston W, McManus R, Pye C, Lewis CM, Cannon-Albright LA, Peto J, Ponder BAJ, Skolnick MH, Easton DF, Goldgar DE and Stratton MR (1994) Localization of a breast cancer susceptibility gene, *BRCA2*, to chromosome 13q12–13. *Science* **265**: 2088–2090

Wooster R, Bignell G, Lancaster J, Swift S, Seal S, Mangion J, Collins N, Gregory S, Gumbs C, Micklem G, Barfoot R, Hamoudi R, Patel S, Rice C, Biggs P, Hashim Y, Smith A, Connor F, Arason A, Gudmundsson J, Ficenec D, Kelsell D, Ford D, Tonin P, Bishop DT, Spurr NK, Ponder BAJ, Eeles R, Peto J, Devilee P, Cornelisse C, Lynch H, Narod S, Lenoir G, Egilsson V, Bjork Barkadottir R, Easton DF, Bentley DR, Futreal PA, Ashworth A and Stratton MR (1995) Identification of the breast cancer susceptibility gene. *BRCA2*. *Nature* **378**: 789–792

 © Cancer Research Campaign 1999

## References

[PDF_00301] Couch F. J., DeShano M. L., Blackwood M. A., Calzone K., Stopfer J., Campeau L., Ganguly A., Rebbeck T., Weber B. L. (1997). BRCA1 mutations in women attending clinics that evaluate the risk of breast cancer.. N Engl J Med.

[PDF_00304] Easton D. F., Bishop D. T., Ford D., Crockford G. P. (1993). Genetic linkage analysis in familial breast and ovarian cancer: results from 214 families. The Breast Cancer Linkage Consortium.. Am J Hum Genet.

[PDF_00307] Hogervorst F. B., Cornelis R. S., Bout M., van Vliet M., Oosterwijk J. C., Olmer R., Bakker B., Klijn J. G., Vasen H. F., Meijers-Heijboer H. (1995). Rapid detection of BRCA1 mutations by the protein truncation test.. Nat Genet.

[PDF_00311] Krainer M., Silva-Arrieta S., FitzGerald M. G., Shimada A., Ishioka C., Kanamaru R., MacDonald D. J., Unsal H., Finkelstein D. M., Bowcock A. (1997). Differential contributions of BRCA1 and BRCA2 to early-onset breast cancer.. N Engl J Med.

[PDF_00321] Miki Y., Swensen J., Shattuck-Eidens D., Futreal P. A., Harshman K., Tavtigian S., Liu Q., Cochran C., Bennett L. M., Ding W. (1994). A strong candidate for the breast and ovarian cancer susceptibility gene BRCA1.. Science.

[PDF_00332] Peelen T., van Vliet M., Petrij-Bosch A., Mieremet R., Szabo C., van den Ouweland A. M., Hogervorst F., Brohet R., Ligtenberg M. J., Teugels E. (1997). A high proportion of novel mutations in BRCA1 with strong founder effects among Dutch and Belgian hereditary breast and ovarian cancer families.. Am J Hum Genet.

[PDF_00335] Petrij-Bosch A., Peelen T., van Vliet M., van Eijk R., Olmer R., Drüsedau M., Hogervorst F. B., Hageman S., Arts P. J., Ligtenberg M. J. (1997). BRCA1 genomic deletions are major founder mutations in Dutch breast cancer patients.. Nat Genet.

[PDF_00327] Petrij-Bosch A., Peelen T., van Vliet M., van Eijk R., Olmer R., Drüsedau M., Hogervorst F. B., Hageman S., Arts P. J., Ligtenberg M. J. (1997). BRCA1 genomic deletions are major founder mutations in Dutch breast cancer patients.. Nat Genet.

[PDF_00347] Shattuck-Eidens D., Oliphant A., McClure M., McBride C., Gupte J., Rubano T., Pruss D., Tavtigian S. V., Teng D. H., Adey N. (1997). BRCA1 sequence analysis in women at high risk for susceptibility mutations. Risk factor analysis and implications for genetic testing.. JAMA.

[PDF_00352] Szabo C. I., King M. C. (1997). Population genetics of BRCA1 and BRCA2.. Am J Hum Genet.

[PDF_00354] Tavtigian S. V., Simard J., Rommens J., Couch F., Shattuck-Eidens D., Neuhausen S., Merajver S., Thorlacius S., Offit K., Stoppa-Lyonnet D. (1996). The complete BRCA2 gene and mutations in chromosome 13q-linked kindreds.. Nat Genet.

[PDF_00371] Wooster R., Bignell G., Lancaster J., Swift S., Seal S., Mangion J., Collins N., Gregory S., Gumbs C., Micklem G. (1995). Identification of the breast cancer susceptibility gene BRCA2.. Nature.

[PDF_00364] Wooster R., Neuhausen S. L., Mangion J., Quirk Y., Ford D., Collins N., Nguyen K., Seal S., Tran T., Averill D. (1994). Localization of a breast cancer susceptibility gene, BRCA2, to chromosome 13q12-13.. Science.

